# Psychometric and structural properties of the Karolinska Exhaustion Disorder Scale: a 1,072-patient study

**DOI:** 10.1186/s12888-023-05138-4

**Published:** 2023-09-02

**Authors:** Elin Lindsäter, Jakob Clason van de Leur, Christian Rück, Erik Hedman-Lagerlöf, Renzo Bianchi

**Affiliations:** 1 Division of Psychology, Department of Clinical Neuroscience, Karolinska Institutet. Gustavsberg Primary Care Clinic, Odelbergs Väg 19, 134 40 Gustavsberg, Sweden; 2 Center for Psychiatry Research, Department of Clinical Neuroscience, Karolinska Institutet, & Stockholm Health Care Services, Region Stockholm, Sweden; 3 Department of Psychology, Uppsala University, Uppsala, Sweden; 4 Department of Psychology, Norwegian University of Science and Technology (NTNU), Trondheim, Norway

**Keywords:** Burnout, Construct validity, Dimensionality, Factor analysis, Health, Psychological stress, Reliability

## Abstract

**Objective:**

Exhaustion disorder is a stress-related diagnosis that was introduced in 2005 to the Swedish version of the International Statistical Classification of Diseases and Related Health Problems, 10^th^ edition (ICD-10). The Karolinska Exhaustion Disorder Scale (KEDS) was developed to assess exhaustion disorder symptomatology. While the KEDS is intended to reflect a single construct and be used based on its total score, the instrument's characteristics have received limited attention. This study investigated the KEDS’s psychometric and structural properties in a large clinical sample.

**Methods:**

The study relied on data from 1,072 patients diagnosed with exhaustion disorder that were included in two clinical trials in Sweden. We investigated the dimensionality, homogeneity, and reliability of the KEDS using advanced statistical techniques, including exploratory structural equation modeling (ESEM) bifactor analysis.

**Results:**

A one-factor confirmatory analytic model exhibited a poor fit, suggesting at least a degree of multidimensionality. The ESEM bifactor analysis found the general factor to explain about 72% of the common variance extracted, with an omega hierarchical coefficient of 0.680. Thus, the ESEM bifactor analysis did not clearly support the scale’s essential unidimensionality. A homogeneity analysis revealed a scale-level *H* of only 0.296, suggesting that KEDS’s total scores do not accurately rank individuals on the latent continuum assumed to underlie the measure. The KEDS’s reliability was modest, signaling considerable measurement error.

**Conclusion:**

Findings reveal important limitations to the KEDS with possible implications for the status of exhaustion disorder as a nosological category.

**Trial registration:**

This study was pre-registered on Open Science Framework (osf.io) on April 24, 2022 (https://osf.io/p34sq/).

**Supplementary Information:**

The online version contains supplementary material available at 10.1186/s12888-023-05138-4.

## Background

Chronic psychosocial stress negatively affects individuals’ health and has high societal costs through reduced work capacity and sickness absence [1, 2]. Based on egalitarian values of social democracy, Sweden has an extensive public health care system and social insurance that aim to be accessible to all citizens on equal terms [3]. In Sweden, stress-related disorders currently account for over 50% of all sickness absences due to mental disorders [4]. In 2005, Exhaustion Disorder (ED) was introduced as a new medical diagnosis into the Swedish version of the International Statistical Classification of Diseases and Related Health Problems (ICD-10; see Table 1 for diagnostic criteria). ED is characterized by persistent mental and physical fatigue believed to develop in the wake of prolonged exposure to intractable stressors [5]. Diagnostic criteria were developed by a task force of researchers commissioned to investigate the rapid increase in sick leave rates in Sweden after a period of economic recession in the late 1990s. Interviews and clinical observations (unpublished to this day) indicated that many individuals on sick leave due to depression presented a clinical picture dominated by fatigue and cognitive complaints and attributed their mental health problems to work-related and psychosocial stressors.

**Table 1:** Diagnostic Criteria for Exhaustion Disorder published by the National Board of Health and Welfare in Sweden

A	Physical and mental symptoms of exhaustion during at least two weeks. The symptoms have developed in response to one or more identifiable stressors, which have been present for at least 6 months	
B	Markedly reduced mental energy, manifested by reduced initiative, lack of endurance, or increased time needed for recovery after mental efforts	
C	At least four of the following symptoms have been present most of the day, nearly every day, during the same 2-week period:	
	1	Persistent complaints of impaired memory and concentration
	2	Markedly reduced capacity to tolerate demands or to perform under time pressure
	3	Emotional instability or irritability
	4	Insomnia or hypersomnia
	5	Persistent complaints of physical fatigue and lack of endurance
	6	Physical symptoms such as muscular pain, chest pain, palpitations, gastrointestinal problems, vertigo, or increased sensitivity to sounds
D	The symptoms cause clinically significant distress or impairment in social, occupational, or other important areas of functioning	
E	The symptoms are not due to the direct physiological effects of a substance (e.g., abuse of a drug or medication) or a general medical condition (e.g., hypothyroidism, diabetes, infectious disease)	
F	If the criteria for major depression, dysthymia, or generalized anxiety disorder are met simultaneously, exhaustion disorder is set only as an additional specification to any such diagnosis	

Note. All criteria with capital letters must be met to set the diagnosis

A recent review of all published empirical ED studies found that research on the validity of this new diagnosis remains limited [6]. The clinical picture of ED is similar to that of burnout [7, 8] and chronic fatigue [9], and the overlap with anxiety and depressive disorders is substantial [6]. Amidst an international debate regarding whether burnout should be conceptualized as a medical disorder [10], ED has not been included in international versions of the ICD or the Diagnostic and Statistical Manual of Mental Disorders (DSM). In Sweden, however, the number of individuals diagnosed with ED has increased rapidly over the years with prevalence estimates approaching those of major depression [11, 12]. Furthermore, ED alone accounts for more long-term sickness absences than any other psychiatric or somatic disorder in the country [4]. Given the functional disability and suffering that can be associated with ED, continued investigation into the new diagnostic construct is merited.

To date, most research on ED has relied on self-rating scales developed to assess burnout, such as the Shirom-Melamed Burnout Questionnaire (SMBQ; [8]). However, several Swedish self-rating scales have been developed to pinpoint ED diagnostic criteria. One of these measures – The Karolinska Exhaustion Disorder Scale (KEDS) – has been widely implemented in clinical practice and workplace settings across Sweden. Despite the popularity of the KEDS, the instrument’s psychometric properties remain largely uninvestigated. In the only published study that has investigated the KEDS’s psychometric properties in a sample of ED patients, principal component analysis reached equivocal results regarding the scale's dimensionality, with the emergence of one- and two-component solutions [13]. A recent Danish study that investigated symptoms of exhaustion in patients with stress-related disorders and other psychiatric and somatic disorders using the KEDS found no difference in total scores between patients diagnosed with major depression and patients diagnosed with a stress-related diagnosis [14]. In addition, the KEDS has been found to correlate only moderately (below 0.50) with other inventories deemed to assess ED symptoms [15]. To further our understanding of the clinical utility of the KEDS, the present study investigated the dimensionality, homogeneity, reliability, and measurement invariance of the KEDS based on data from a large ED sample.

## Methods

### Study procedure and sampling

The KEDS was evaluated using baseline self-ratings from 1,072 patients diagnosed with ED that were either (1) included in an open clinical trial of specialized multimodal rehabilitation for ED at two clinics in Stockholm, Sweden, between September 2017 and March 2019 (*n* = 914) [16, 17] or (2) included in a randomized clinical trial of Internet-delivered treatment for stress-related disorders (ClinicalTrials.gov Identifier: NCT04797273) between April 2021 and April 2022 (*n* = 158). In the open clinical trial, multi-professional teams (physician, psychologist, and physiotherapist) assessed each patient referred for ED before confirming the ED diagnosis. In the randomized trial, a national sample of self-referred participants underwent structured clinical assessment before inclusion to the study, where the ED diagnosis was set. Ethical approvals were obtained, and all participants signed informed consent. Table 2 presents participant characteristics of the total sample. The pre-registered analysis plan for the present study is available at Open Science Framework (osf.io), https://osf.io/p34sq/.

**Table 2:** Baseline characteristics of patients diagnosed with exhaustion disorder included in the study

Baseline characteristics	Total *N* = 1,072			
	*n*	%	Mean	SD
Age			43.27	9.35
Sex, female	965	87		
Marital status, partner	770	72		
Marital status, single	302	28		
Education level, low	31	3		
Education level, moderate	232	22		
Education level, high	809	75		
Employed/self-employed	954	89		
No sick leave	345	32		
Part-time sick leave	303	28		
Full-time sick leave	424	40		
Baseline KEDS-scores			34.41	6.32

Note. *KEDS* Karolinska Exhaustion Disorder Scale

### Measure of interest

The KEDS is a symptom self-rating scale developed to screen for ED cases and evaluate symptom progression. The items in the KEDS were selected from the Comprehensive Psychopathological Rating Scale [18] based on their correspondence with ED diagnostic criteria, and complemented with items developed based on clinical reports from ED patients [13]. A detailed description of the development of the KEDS is provided by Besèr et al. [13] and the English version of the scale is presented in the Supplementary Material. The KEDS comprises nine items that cover (1) ability to concentrate, (2) memory, (3) physical stamina, (4) mental stamina, (5) recovery, (6) sleep, (7) hypersensitivity to sensory impressions, (8) experience of demands, and (9) irritation and anger. Responses to each item are given on a seven-point scale (0–6) with short descriptive phrases provided on the scale’s rating points 0, 2, 4, and 6. The sum score is 0–54, with higher values reflecting more severe symptoms.

Descriptive statistics of the KEDS in the present sample are displayed in Table 3.

**Table 3:** Descriptive statistics (*N* = 1,072)

	KEDS1	KEDS2	KEDS3	KEDS4	KEDS5	KEDS6	KEDS7	KEDS8	KEDS9	KEDS
Mean	3.746	3.782	3.355	3.882	4.493	3.873	3.936	4.027	3.317	3.823
Standard deviation	1.045	1.282	1.080	0.904	1.106	1.471	1.403	0.919	1.398	0.703
Median	4	4	3	4	4	4	4	4	4	3.889
Interquartile range	1	2	1	0	1	2	2	1	2	0.889
Mode	4	3	4	4	4	4	4	4	4	3.556
Skewness (*SE* = 0.075)	-0.665	0.075	-0.346	-0.554	-0.218	-0.293	-0.564	-0.321	-0.532	-0.124
Kurtosis (*SE* = 0.149)	0.676	-0.391	0.180	0.542	-0.689	-0.603	0.253	0.572	-0.045	0.048
Minimum	0	0	0	1	1	0	0	1	0	1.556
Maximum	6	6	6	6	6	6	6	6	6	6.000

Notes. *SE* Standard error, *KEDS* Total score on the Karolinska Exhaustion Disorder Scale, *KEDS1* Concentration item, *KEDS2* Memory item, *KEDS3* Physical stamina item, *KEDS4* Mental stamina item, *KEDS5* Recovery item, *KEDS6* Sleep item, *KEDS7* Sensory hypersensitivity item, *KEDS8* Experience of demands item, *KEDS9* Irritation/anger item

KEDS-related scores were generally negatively skewed, which is expected in a clinical sample such as ours. The most commonly endorsed item was item 5 (recovery), and the least commonly endorsed item was item 9 (irritation/anger). The mean Pearson correlation among the nine KEDS items was 0.285 (*SD* = 0.093).

### Data analyses

We conducted our factor analyses in Mplus 8.6 [19]. In all factor analyses, we treated the items as ordinal, relied on the weighted least squares—mean and variance adjusted—estimator, and focused on the following fit indices: the Root Mean Square Error of Approximation (RMSEA; cutoff: 0.080), the Comparative Fit Index (CFI; cutoff: 0.950), the Tucker-Lewis Index (TLI; cutoff: 0.950), and the Standardized Root Mean Squared Residual (SRMR; cutoff: 0.080). We first investigated the dimensionality of the KEDS based on confirmatory factor analysis (CFA). Clarifying the dimensionality of a scale is important to establish the usability of the scale’s total score. Because the KEDS is assumed to capture a unidimensional construct—exhaustion disorder—we examined a one-factor model.

**Fig. 1:**
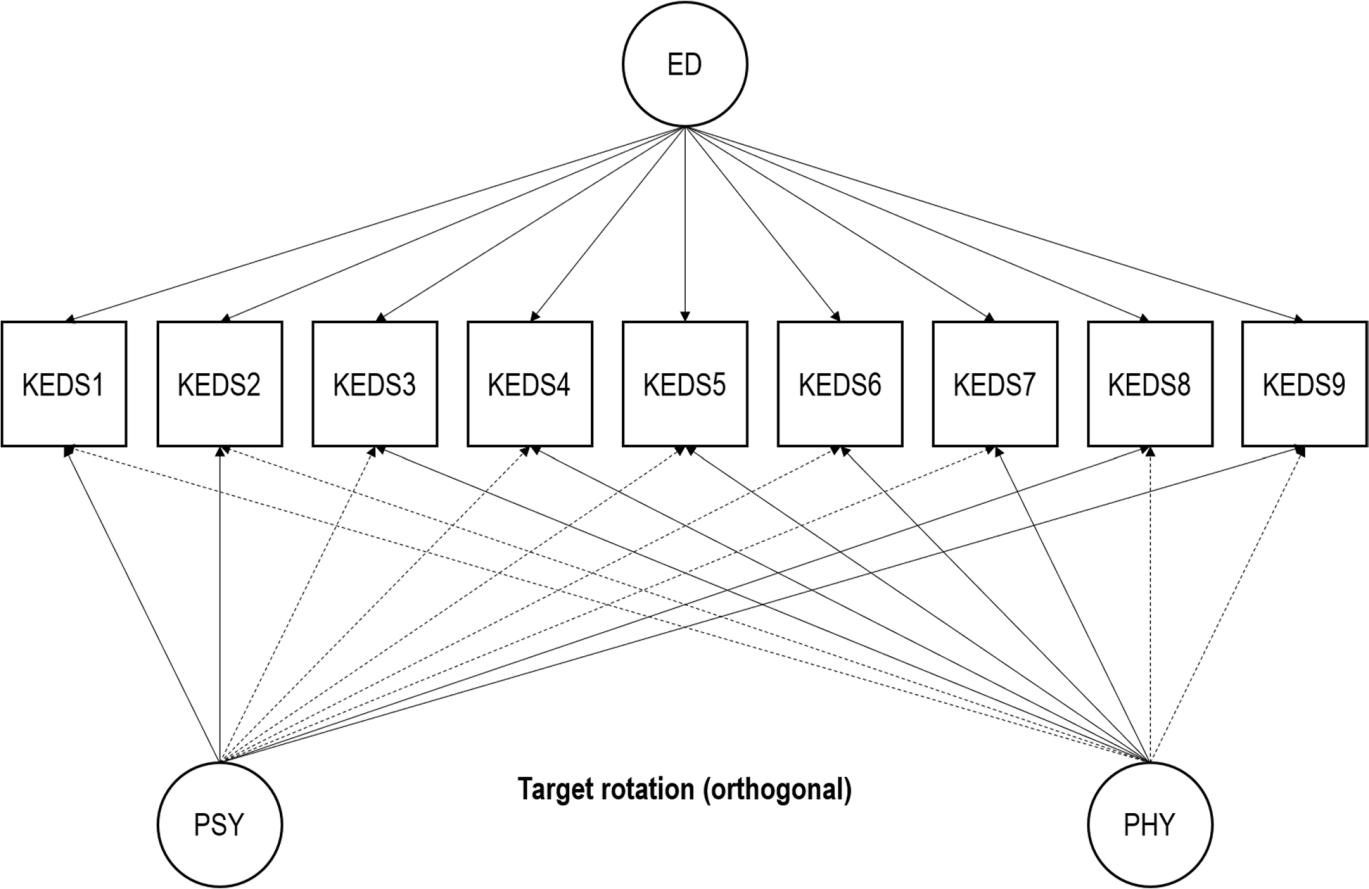
Illustration of the exploratory structural equation modeling bifactor structure under examination. The dashed arrows indicate nontarget loadings. *ED* General Exhaustion Disorder factor, *PSY* Psychological bifactor, *PHY* Physical bifactor, *KEDS1* Concentration item, *KEDS2* Memory item, *KEDS3* Physical stamina item, *KEDS4* Mental stamina item, *KEDS5* Recovery item, *KEDS6* Sleep item, *KEDS7* Sensory hypersensitivity item, *KEDS8* Experience of demands item; KEDS9: irritation/anger item

Envisaging that the KEDS may involve a degree of multidimensionality due to its inclusion of both psychological and physical symptom items, we further inquired into the instrument’s dimensionality using exploratory structural equation modeling (ESEM) bifactor analysis [20]. ESEM bifactor analysis is particularly helpful in ascertaining whether a measure involving a degree of multidimensionality is nevertheless “unidimensional enough” for the measure’s total score to be justifiably used [21]. We employed a target rotation allowing us to adopt a confirmatory approach [20, 22]. An advantage of the target rotation is that nontarget loadings are not fixed to be equal to 0; instead, they are “encouraged” to get as close to 0 as possible by the loss function, allowing factorial complexity to be modeled. We extracted two specific factors (or bifactors) in addition to the general Exhaustion Disorder factor on account of the psychological and physical symptom items populating the KEDS. The Psychological bifactor targeted items 1 (concentration), 2 (memory), 8 (experience of demands), and 9 (irritation/anger); the Physical bifactor target items 3 to 7 (i.e., physical stamina, mental stamina, recovery, sleep, and sensory hypersensitivity). A graphical illustration of the model tested is displayed in Fig. 1.

To estimate the proportion of the common variance explained by the general factor in our ESEM bifactor analysis, we computed explained common variance (ECV) indices. A scale-level ECV index of at least 0.800 is suggestive of essential unidimensionality. We additionally computed the Omega Hierarchical (OmegaH) coefficient. OmegaH estimates the proportion of variance in total scores that can be attributed to the general factor, thus treating variability in scores due to specific factors as measurement error [21, 23].

We investigated the KEDS’s homogeneity using the Mokken package version 3.0.6 [24] in R version 4.0.3 (R Core Team, 2020). This analysis was conducted post-hoc to deepen the understanding of the scale’s properties. Homogeneity analysis relies on Loevinger’s *H* coefficient, which provides information on the degree to which a scale’s total score accurately ranks individuals on a latent continuum [25]. Homogeneity is considered to be strong when *H* ≥ 0.50, moderate when 0.40 ≤ *H* < 0.50, and weak when 0.30 ≤ *H* < 0.40. A scale-level *H* < 0.30 suggests that (a) the measure of interest cannot be regarded as unidimensional and (b) a total-score approach is likely ill-advised [25, 26].

We used the same R package to investigate the KEDS’s total-score reliability. We focused on Cronbach's alpha, Guttman’s lambda-2, and the Molenaar-Sijtsma statistic. As recommended in the context of basic research, we considered a threshold of 0.80 (rather than 0.70) as a minimum for satisfactory reliability [27, 28].

Lastly, we examined the measurement invariance of a unidimensional model across sexes (male/female) and age groups (based on a median spit). As previously noted, our sample included 965 women and 107 men. The mean age was 43.27 (*SD*_AGE_ = 9.35); our median split resulted in a younger group (< 44 years) of 557 patients and an older group (≥ 44 years) of 515 patients. As can be seen from Table 3, three KEDS items (items 4, 5, and 8) had scores ranging from 1 to 6; put differently, no respondent selected a score of 0 on these items. To be able to conduct the measurement invariance analysis, we recoded scores of 0 into scores of 1 for the remaining six items, resulting in a total of 117 replacements.^1^ In our analysis, we focused on: (a) configural invariance—the equivalence of overall factorial structures; (b) metric invariance—the equivalence of factor loadings; and (c) scalar invariance—the equivalence of item thresholds [29]. As recommended, invariance violations were flagged based on thresholds of -0.010 for changes in CFI and TLI, and 0.015 for changes in RMSEA [30–32].

## Results

### Confirmatory factor analysis

A one-factor model exhibited a poor fit: RMSEA = 0.099; CFI = 0.932; TLI = 0.910; SRMR = 0.060; *χ*^*2*^ (27) = 311.061. Factor loadings ranged from 0.388 for the irritation/anger item to 0.721 for the mental stamina item (*M* = 0.574, *SD* = 0.119). Residual variances ranged from 0.480 for the mental stamina item to 0.849 for the irritation/anger item.

### ESEM bifactor analysis

The specified ESEM bifactor structure showed an acceptable fit: RMSEA = 0.065; CFI = 0.987; TLI = 0.961; SRMR = 0.016; *χ*^*2*^ (12) = 66.276. All items loaded > 0.300 on the general Exhaustion Disorder factor (*M* = 0.538, *SD* = 0.130), but only one item (experience of demands) displayed a factor loading exceeding 0.700. The Physical bifactor was relatively well-delineated; this was less the case for the Psychological bifactor. The scale-level ECV index had a value of 0.716, indicating that the general factor accounted for about 72% of the common variance extracted. Factor loadings and ECV indices are available in Table 4. OmegaH was 0.680, indicating that a substantial proportion of the variance in total scores could *not* be attributed to the general factor.

**Table 4:** Exploratory structural equation modeling bifactor analysis of the Karolinska Exhaustion Disorder Scale (KEDS)

Items	ED (ʎ)	PSY (ʎ)	PHY (ʎ)	I-ECV
KEDS1—concentration	0.650	0.332	0.102	0.778
KEDS2—memory	0.553	0.504	-0.040	0.545
KEDS3—physical stamina	0.378	-0.123	0.487	0.362
KEDS4—mental stamina	0.638	-0.022	0.385	0.733
KEDS5—recovery	0.553	0.114	0.489	0.547
KEDS6—sleep	0.374	0.087	0.290	0.603
KEDS7—sensory hypersensitivity	0.503	-0.058	0.106	0.944
KEDS8—experience of demands	0.755	-0.185	0.029	0.942
KEDS9—irritation/anger item	0.435	-0.001	-0.052	0.986

Notes. *N* = 1,072. ʎ Factor loading, *ED* General Exhaustion Disorder factor, *PSY* Psychological bifactor, *PHY* Physical bifactor, *I-ECV* Item-level explained common variance index. The scale-level explained common variance index was 0.716; subscale-level explained common variance indices were 0.813 for the PSY items and 0.638 for the PHY items

### Homogeneity and total-score reliability

The scale-level *H* coefficient was 0.296 (*SE* = 0.014; 95% CI = 0.268–0.323), meaning that the KEDS total score did not accurately rank individuals on a latent continuum. Using the automated item selection procedure, we found items 3, 6, and 9 (physical stamina, sleep, and irritation/anger) to be unscalable at the threshold of 0.296. These findings suggest that the scale cannot be considered unidimensional and that a total-score approach is likely ill-advised in practice (see Supplementary Material Tables S1 and S2 for more granular information).

Cronbach's alpha had a value of 0.763. Both Guttman’s lambda-2 and the Molenaar-Sijtsma statistic reached 0.765, below our threshold of 0.80 as a minimum for satisfactory reliability. The obtained coefficients thus signaled considerable measurement error, suggesting problematic total-score reliability.

### Measurement invariance analysis

Upon preregistration, the measurement invariance analysis of the KEDS was planned to target a unidimensional model. Because our results showed that the scale can barely be regarded as unidimensional, results from this analysis are reported indicatively in the Supplementary Material (Table S3). In brief, the fit indices did not deteriorate as we added constraints from configural to metric to scalar models, suggesting invariance across sexes and age groups.

## Discussion

The present study examined the psychometric and structural properties of the KEDS in a sample of 1,072 ED patients. While the KEDS was designed to capture a unitary construct, results from our CFA and ESEM bifactor analysis provided little support for the scale’s essential unidimensionality. Consistent with our factor analytic findings, a homogeneity analysis indicated that the use of the KEDS’s total score is likely ill-advised. One-third of the items did not align well on the latent continuum assumed to underlie the measure. Furthermore, the total-score reliability of the instrument was modest, with values in the 0.70 s. Such values, which reveal considerable measurement error, are considered insufficient for scales meant to be used in basic research, and clearly problematic for instruments intended to be employed in applied settings [27, 28].

The limited internal consistency of the KEDS found in our study aligns with findings from the only previous psychometric evaluation of the KEDS among ED patients, conducted by Besèr et al. [13]. Such a finding is a cause for concern if the KEDS is to be used in applied settings involving clinical decision-making (e.g., for granting sick leave or prescribing treatment). Examining the latent structure of the KEDS with principal component analysis, Besér et al. obtained equivocal results. However, the authors concluded that a one-component structure best described the scale. In our study, we did not find evidence that the KEDS can be considered unidimensional. The divergent conclusions from the two studies might be due to variations in the statistical methods used, and the small sample size (*N* = 200) in the study of Besér et al. as compared with our large sample size (*N* = 1,072). It may also be that the samples used in the two studies represent somewhat different patient populations. ED patients in the study by Besèr et al. were recruited at a time when the ED diagnosis was recent in Sweden (2005–2010), whereas patients in our study were recruited between 2017 and 2022. Between the years of 2005 and 2022, the use of the ED diagnosis has increased markedly [4]. Even though the sociodemographic data indicate that the topographic qualities of the two samples are similar (middle-aged, highly educated women on sick leave), it is plausible that there, over the years, has been a shift in diagnostic trends such that some patients that would previously have been diagnosed with, for example, major depression or anxiety disorders where mental fatigue is a common symptom [33, 34], have instead been diagnosed with ED. In other words, it is conceivable that the patient group diagnosed with ED has become more heterogeneous with time. In Sweden, the recommendations for sick leave provided by the Swedish Social Insurance Agency stipulate that ED patients may be granted sick leave reimbursement for up to one year, whereas patients with other common mental disorders (e.g., depression) are usually eligible for part-time sick leave periods of 3 to 6 months. Such differentiated regulations might trigger healthcare professionals to use the ED diagnosis for a range of fatigued patients suffering from functional impairment.

There are at least two potential explanations of our findings. First, it might be that ED indeed constitutes a unitary diagnostic construct and that the KEDS simply fails to assess ED properly. The advanced statistical techniques employed in our study allow investigators to ascertain whether a scale can be used based on its total score despite the presence of a degree of multidimensionality. Measures such as the PHQ-9, a depression scale that covers as many as nine symptoms, have been found to meet such requirements in both clinical and nonclinical samples [35, 36].

A second way of interpreting our results is that, rather than dealing with an *operationalization* problem (i.e., a problem at the level of the instrument), we are dealing with a *conceptualization* problem. A conceptualization problem would imply that the diagnostic construct of ED is poorly devised, rendering any measures of the construct psychometrically unsound. Conceptualization issues would bear on the very definition and clinical validity of ED. As mentioned previously, ED is a relatively new medical diagnosis that has only been accepted into the Swedish version of the ICD-10. Very few studies have investigated the clinical validity and the specificity of ED in relation to other diagnostic constructs [6]. It is, however, well-established that the core symptom of ED (exhaustion/fatigue) is common to a range of psychiatric and somatic disorders [33, 37], and is a widespread experience in the general population [38]. The large symptom overlap of ED with anxiety and depressive disorders found in previous studies [6], together with the wide range of somatic symptoms expressed by ED patients [39, 40], suggest that any measure attempting to capture ED diagnostic criteria risks masking considerable multimorbidity.

Given the limited knowledge regarding ED as a diagnostic construct, and the finding that the KEDS may lack psychometric and structural robustness when employed with ED patients, a future avenue to further the understanding of ED might be to focus on the core symptom, namely, fatigue. Several internationally acknowledged fatigue self-rating scales have been found to be unidimensional with good reliability and validity [41]. The use of such scales could increase the comparability and integration of results across (clinical) samples, trials, and countries. This, in turn, could promote knowledge accumulation regarding the Swedish ED diagnosis and its relation to other fatigue-dominated conditions.

### Limitations

The present study has limitations. First, the use of a cross-sectional design prevented us from examining properties such as test–retest reliability and temporal measurement invariance. Second, our study was centered on “intrinsic” properties of the KEDS and did not examine the scale’s discriminant validity vis-à-vis measures of related constructs (such as anxiety or depression). Because construct proliferation has become a concern in psychological and medical sciences [42–44], an examination of the KEDS’s discriminant validity would have been an added advantage. Third, the current study assessed the psychometric and structural properties of the KEDS in a clinical sample, and the obtained findings may not generalize to the general population. Fourth, generalizability might be further limited by the fact that study participants were almost exclusively ethnic Swedes. Results should be replicated using a population-based sample**.**

## Conclusions

This study investigated the psychometric and structural properties of the KEDS in a large sample of patients diagnosed with ED. Results indicate that the scale does not meet the requirements for essential unidimensionality and lacks reliability, suggesting that using the KEDS’s total score for the assessment and screening of ED may be ill-advised. Because the KEDS closely adheres to the current diagnostic criteria for ED, our findings may have implications for ED as a nosological and diagnostic category. The sturdiness of ED as a pathological entity requires further investigation.

### Supplementary Information

**Additional file 1.**

## Data Availability

The datasets generated and/or analyzed during the current study are not publicly available (to protect the privacy of study participants) but are available from the corresponding author on reasonable request. Code used for analyses can be accessed by contacting the last author, RB.

## List of abbreviations

CFA: Confirmatory factor analysis
CFI: Comparative Fit Index
ECV: Explained common variance
ED: Exhaustion disorder
ESEM: Exploratory structural equation modeling
KEDS: Karolinska Exhaustion Disorder Scale
OmegaH: Omega Hierarchical
RMSEA: Root Mean Square Error of Approximation
SRMR: Standardized Root Mean Squared Residual
TLI: Tucker-Lewis Index

## Footnotes

1 Seven for item 1; six for item 2; ten for item 3; fourteen for item 6; twenty-nine for item 7; and fifty-one for item 9.
