# Supplementary material for: Psychometric and structural properties of the Karolinska Exhaustion Disorder Scale: a 1,072-patient study
Source: BMC Psychiatry. 2023 Sep 2;23:642. doi: 10.1186/s12888-023-05138-4 (PMC10475192; doi:10.1186/s12888-023-05138-4)
Supplement: Supplementary file 1 — Additional file 1. [file 12888_2023_5138_MOESM1_ESM.docx]

# Supplementary material

#

**Karolinska Exhaustion Disorder Scale**

English translation published in Besèr, A., Sorjonen, K., Wahlberg, K., Peterson, U., Nygren, Å., & Åsberg, M. (2014). Construction and evaluation of a self rating scale for stress‐induced exhaustion disorder, the Karolinska Exhaustion Disorder Scale. *Scandinavian Journal of Psychology, 55*(1), 72-82. <https://doi.org/10.1111/sjop.12088>

*Instructions*

The purpose of this form is to provide an overall picture of your current (physical/emotional) state. We would like you to try to rate how you have been feeling during the past two weeks.

This form contains a series of statements about how one can feel in several different respects. These statements express different degrees of uneasiness, from lack of discomfort to a maximum and pronounced feeling of unease.

Draw a cross in the square in front of the number that you think corresponds best to the way you have been feeling the past two weeks.

1. **Ability to concentrate**

We would like you to assess your ability to keep your thoughts together and concentrate on various activities. Think about how you function in various activities that demand different levels of concentration, e.g., reading a complicated text, reading a newspaper article and watching TV.

|  | 0 | I do not have any difficulty concentrating, and can read, watch TV and converse normally. |
| --- | --- | --- |
|  | 1 |  |
|  | 2 | I occasionally have difficulty keeping my thoughts together on things that would normally hold my attention. |
|  | 3 |  |
|  | 4 | I have often difficulty concentrating. |
|  | 5 |  |
|  | 6 | I cannot concentrate on anything at all. |

1. **Memory**

We ask here that you describe your ability to remember things. Think about whether or not you have had difficulty recalling names, dates, or tasks that you intend to do during a regular day.

|  | 0 | I remember names, dates, and what I am supposed to do. |
| --- | --- | --- |
|  | 1 |  |
|  | 2 | Sometimes I forget things that are not so important, but if I pull myself together, I can usually remember. |
|  | 3 |  |
|  | 4 | I often forget appointments or names of people whom I know very well. |
|  | 5 |  |
|  | 6 | Every day, I forget important things or what I have promised to do. |

1. **Physical stamina**

This is a question concerning your physical stamina. Do you feel, for example, more exhausted than usual after the activities of an ordinary day or some form of physical exertion?

|  | 0 | I feel the way I usually do and perform my daily physical activities or exercise as usual. |
| --- | --- | --- |
|  | 1 |  |
|  | 2 | I feel that physical effort is more exhausting than normal, but still move the way I usually do in this respect. |
|  | 3 |  |
|  | 4 | I do not have the energy to exert myself physically. It is OK as long as I move at a normal phase, but I cannot increase my pace without becoming shaky and short of breath. |
|  | 5 |  |
|  | 6 | Every day, I forget important things or what I have promised to do. |

1. **Mental stamina**

We would like you to reflect here on your mental stamina and to what extent you are more mentally exhausted than usual in various everyday situations.

|  | 0 | I have just as much energy as usual. I do not have any particular difficult performing my daily activities. |
| --- | --- | --- |
|  | 1 |  |
|  | 2 | I can manage my everyday activities, but they take more energy, and I am exhausted more quickly than usual. I need to take breaks more often than usual. |
|  | 3 |  |
|  | 4 | I become inordinately tired when I attempt my daily activities and find social situations exhausting. |
|  | 5 |  |
|  | 6 | I do not have the energy to do anything. |

1. **Recovery**

We ask you to describe here how well and how quickly you recover mentally and physically when you have been exhausted.

|  | 0 | I do not have to rest during the day. |
| --- | --- | --- |
|  | 1 |  |
|  | 2 | I become tired during the day, but all I have to do is to take a little break in order to recover. |
|  | 3 |  |
|  | 4 | I become tired during the day and need to take long breaks in order to feel fit. |
|  | 5 |  |
|  | 6 | No matter how much I rest, it feels as if I am unable to recharge my batteries. |

1. **Sleep**

We ask you to describe your sleep. Think about how long you have slept and the quality of your sleep during the past two weeks. Your assessment should reflect your actual sleep, regardless of whether or not you have taken sleeping pills.

|  | 0 | I sleep well and long enough. I usually feel thoroughly rested when I wake up after a night’s sleep. |
| --- | --- | --- |
|  | 1 |  |
|  | 2 | Sometimes, I sleep more restlessly than usual, or wake up during the night and have difficulty going back to sleep. Sometimes, I do not feel thoroughly rested when I wake up after a night’s sleep. |
|  | 3 |  |
|  | 4 | I often sleep more restlessly than usual or wake up during the night and have difficulty going back to sleep. I often have a feeling of not being thoroughly rested after a night’s sleep. |
|  | 5 |  |
|  | 6 | I sleep superficially or restlessly every night. I never feel thoroughly rested after a night’s sleep. |

1. **Hypersensitivity to sensory impressions**

This is a question about the extent to which one or several of your senses have become more sensitive to impressions, such as sound, light, smell or touch.

|  | 0 | I do not think that my senses are more sensitive than usual. |
| --- | --- | --- |
|  | 1 |  |
|  | 2 | Sound or light or other sensory impressions are sometimes unpleasant. |
|  | 3 |  |
|  | 4 | I often experience that sound, light or other sensory impressions are disturbing or unpleasant. |
|  | 5 |  |
|  | 6 | Sound, light or other sensory impressions bother me so much that I withdraw in order to give my senses a chance to rest. |

1. **Experience of demands**

Here we ask you to give expression to the way you react to demands in your daily life. These demands can come from your surroundings or be your own demands on yourself.

|  | 0 | I do what I am supposed to do or want to do without experiencing it as especially demanding or difficult. |
| --- | --- | --- |
|  | 1 |  |
|  | 2 | Sometimes I experience daily situations that I used to handle without any particular problem as demanding, leading to unease, or causing me to become more easily stressed. |
|  | 3 |  |
|  | 4 | I often feel that situations that I previously handled without problem are now demanding and cause a strong feeling of uneasiness or stress. |
|  | 5 |  |
|  | 6 | l experience nearly everything as demanding and cannot handle it at all. |

1. **Irritation and anger**

This question regards how easily irritated or angry you become, regardless of whether or not you show it. Think especially about how quick tempered you have been in relationship to the source of your irritation, and how often and intensively you have become angry or irritated. If you have not had any such feelings at all, then you should mark “0.”

|  | 0 | I do not feel that I am especially easily irritated. |
| --- | --- | --- |
|  | 1 |  |
|  | 2 | I am more impatient and easily irritated than usual, but the feeling quickly passes. |
|  | 3 |  |
|  | 4 | I become more impatient and easily irritated than usual. Sometimes I lose control in a way that is unusual for me. |
|  | 5 |  |
|  | 6 | I am often furious and have to make an enormous effort in order to restrain myself. |

| **Supplementary Table S1.** Pairwise *H* coefficients. | | | | | | | | | | | | | | | | |
| --- | --- | --- | --- | --- | --- | --- | --- | --- | --- | --- | --- | --- | --- | --- | --- | --- |
|  | KEDS2 | *SE* | KEDS3 | *SE* | KEDS4 | *SE* | KEDS5 | *SE* | KEDS6 | *SE* | KEDS7 | *SE* | KEDS8 | *SE* | KEDS9 | *SE* |
| KEDS1 | 0.529 | 0.03 | 0.231 | 0.037 | 0.439 | 0.032 | 0.413 | 0.031 | 0.298 | 0.032 | 0.296 | 0.031 | 0.417 | 0.034 | 0.217 | 0.033 |
| KEDS2 | — | — | 0.143 | 0.035 | 0.326 | 0.035 | 0.346 | 0.029 | 0.213 | 0.033 | 0.242 | 0.035 | 0.315 | 0.033 | 0.242 | 0.034 |
| KEDS3 |  |  | — | — | 0.455 | 0.03 | 0.386 | 0.029 | 0.274 | 0.033 | 0.295 | 0.031 | 0.302 | 0.034 | 0.105 | 0.033 |
| KEDS4 |  |  |  |  | — | — | 0.545 | 0.028 | 0.272 | 0.033 | 0.322 | 0.031 | 0.485 | 0.031 | 0.205 | 0.034 |
| KEDS5 |  |  |  |  |  |  | — | — | 0.388 | 0.031 | 0.315 | 0.031 | 0.416 | 0.03 | 0.197 | 0.033 |
| KEDS6 |  |  |  |  |  |  |  |  | — | — | 0.192 | 0.032 | 0.265 | 0.033 | 0.219 | 0.032 |
| KEDS7 |  |  |  |  |  |  |  |  |  |  | — | — | 0.367 | 0.032 | 0.232 | 0.035 |
| KEDS8 |  |  |  |  |  |  |  |  |  |  |  |  | — | — | 0.305 | 0.033 |
| *Notes.* KEDS1: concentration item; KEDS2: memory item; KEDS3: physical stamina item; KEDS4: mental stamina item; KEDS5: recovery item; KEDS6: sleep item; KEDS7: sensory hypersensitivity item; KEDS8: experience of demands item; KEDS9: irritation/anger item; *SE*: standard deviation. | | | | | | | | | | | | | | | | |

| **Supplementary Table S2.** Item-level *H* coefficients. | | | |
| --- | --- | --- | --- |
| Item | Item *H* | *SE* | 95% CI |
| KEDS1 – Concentration | 0.347 | 0.018 | [0.311,0.382] |
| KEDS2 – Memory | 0.285 | 0.019 | [0.247,0.323] |
| KEDS3 – Physical stamina | 0.262 | 0.019 | [0.225,0.299] |
| KEDS4 – Mental stamina | 0.366 | 0.017 | [0.332,0.400] |
| KEDS5 – Recovery | 0.363 | 0.016 | [0.331,0.395] |
| KEDS6 – Sleep | 0.260 | 0.019 | [0.222,0.297] |
| KEDS7 – Sensory hypersensitivity | 0.273 | 0.019 | [0.236,0.310] |
| KEDS8 – Experience of demands | 0.351 | 0.019 | [0.314,0.387] |
| KEDS9 – Irritation/anger | 0.215 | 0.021 | [0.174,0.255] |
| *Notes.* KEDS1: concentration item; KEDS2: memory item; KEDS3: physical stamina item; KEDS4: mental stamina item; KEDS5: recovery item; KEDS6: sleep item; KEDS7: sensory hypersensitivity item; KEDS8: experience of demands item; KEDS9: irritation/anger item; *SE*: standard deviation; 95% CI: 95% confidence interval. | | | |

| **Supplementary Table S3.** Measurement invariance analysis across sexes and age groups. | | | | | | | | | | |
| --- | --- | --- | --- | --- | --- | --- | --- | --- | --- | --- |
| Invariance model | *χ²* | df | RMSEA | CFI | TLI | ΔRMSEA | ΔCFI | ΔTLI | Comp. |  |
| Sexes |  |  |  |  |  |  |  |  |  |  |
| 1. Configural | 330.849 | 54 | .098 | .933 | .911 | — | — | — | — |  |
| 2. Metric | 288.517 | 62 | .083 | .945 | .936 | -.015 | .012 | .025 | M1 |  |
| 3. Scalar | 334.346 | 97 | .068 | .943 | .957 | -.015 | -.002 | .021 | M2 |  |
| Age groups |  |  |  |  |  |  |  |  |  |  |
| 1. Configural | 370.898 | 54 | .105 | .925 | .900 | — | — | — | — |  |
| 2. Metric | 318.871 | 62 | .088 | .939 | .929 | -.017 | .014 | .029 | M1 |  |
| 3. Scalar | 345.236 | 97 | .069 | .941 | .956 | -.019 | .002 | .027 | M2 |  |
| *Notes.* RMSEA: Root Mean Square Error of Approximation; CFI: Comparative Fit Index; TLI: Tucker-Lewis Index | | | | | | | | | | |
